# Single-incision robotic surgery: the emerging milestone in minimally invasive practice

**DOI:** 10.1097/MS9.0000000000004603

**Published:** 2025-12-15

**Authors:** Hasibullah Aminpoor, Muhammad Khizar, Muhammad Zaib, Abeer Ijaz, Hasiba Karimi

**Affiliations:** a Faculty of Medicine, Kabul University of Medical Sciences “Abu Ali Ibn Sina”, Kabul, Afghanistan; b Faculty of Medicine, Georgian American University, Manifest Medical Research Co, Tbilisi, Georgia; c Faculty of Medicine, SMBBMCL Karachi, Karachi, Pakistan; d Faculty of Medicine, Bezmialem Vakif University, Istanbul, Turkey

**Keywords:** da Vinci SP system, minimally invasive surgery, robotic-assisted surgery, single-incision robotic surgery, single-port surgery, Versius robotic system

## Abstract

Single-incision robotic surgery (SIRS) has emerged as a progression in minimally invasive practice, enabling complex operations through a single entry point. Modern systems such as the da Vinci SP (Intuitive Surgical, USA) and Versius (CMR Surgical, UK) allow multi-instrument articulation and high-definition visualization through a single port, overcoming the ergonomic and triangulation challenges of traditional laparoscopy. Recent evidence from South Korea, China, the USA, and Turkey demonstrates SIRS’s feasibility, safety, and promising clinical outcomes across specialties including urology, gynecology, and general surgery. Studies report lower conversion rates, minimal postoperative pain, and enhanced cosmesis, though cost and learning curve remain limiting factors. As robotic technologies evolve, SIRS is poised to redefine minimally invasive standards through precision, reduced invasiveness, and improved recovery profiles. Rigorous multicenter trials and cost-effectiveness analyses are essential to confirm its broader clinical value.

*To the Editor,*

Single-incision robotic surgery (SIRS) represents a major milestone in the evolution and development of minimally invasive techniques. By integrating robotic articulation within a single access point, SIRS provides enhanced dexterity, stable visualization, and improved ergonomics compared to conventional single incision laparoscopy^[1]^. The da Vinci SP and Versius systems exemplify this innovation, utilizing three wristed instruments and an endoscope through a 25–30 mm incision. Such technological precision facilitates intricate operations with minimal tissue disruption and improved aesthetic outcomes, which can be utilized beyond benefits.

Recent global evidence underscores its growing acceptance. In South Korea, robotic single-site cholecystectomy demonstrated excellent safety and ergonomic advantages over conventional single-site laparoscopy^[2]^. A 2025 meta-analysis from China involving over 75 000 patients reported lower conversion rates and equivalent complication profiles for single-incision robotic cholecystectomy compared with traditional approaches^[3]^. Similarly, a U.S. study confirmed that single-incision robotic colectomy yields comparable results to multiport laparoscopy, validating its feasibility in colorectal surgery^[4]^. Beyond general surgery, single-incision robotic hysterectomy and adrenalectomy have proven safe and reproducible, with Turkish studies confirming their practicality and reduced postoperative morbidity^[5]^. Although early costs and operative times remain higher, ongoing refinements in robotic design and surgeon training promise to mitigate these challenges. The continued expansion of SIRS across surgical domains exemplifies precision-driven innovation aimed at enhancing patient recovery, minimizing scarring, and improving surgeon ergonomics. Future directions include standardizing training pathways and establishing long-term outcome registries. Our work is in line with the TITAN Guidelines on the need for transparency in AI use in healthcare^[6]^.

## Data Availability

Not applicable.
